# Cardiac Conduction Safety during Coadministration of Artemether-Lumefantrine and Lopinavir/Ritonavir in HIV-Infected Ugandan Adults

**DOI:** 10.1155/2011/393976

**Published:** 2011-04-07

**Authors:** Pauline Byakika-Kibwika, Mohammed Lamorde, Peter Lwabi, Wilson B. Nyakoojo, Violet Okaba-Kayom, Harriet Mayanja-Kizza, Marta Boffito, Elly Katabira, David Back, Saye Khoo, Concepta Merry

**Affiliations:** ^1^Infectious Diseases Institute, Makerere University, P.O. Box 22418, Kampala, Uganda; ^2^Trinity College, Dublin 2, Ireland; ^3^Infectious Diseases Network for Treatment and Research in Africa (INTERACT), P.O. Box 7062, Kampala, Uganda; ^4^Uganda Heart Institute, Mulago Hospital, P.O. Box 7051, Kampala, Uganda; ^5^St. Stephen's AIDS Trust, London SW10 9TR, UK; ^6^University of Liverpool, Liverpool L69 3BX, UK

## Abstract

*Background*. We aimed to assess cardiac conduction safety of coadministration of the CYP3A4 inhibitor lopinavir/ritonavir (LPV/r) and the CYP3A4 substrate artemether-lumefantrine (AL) in HIV-positive Ugandans. *Methods*. Open-label safety study of HIV-positive adults administered single-dose AL (80/400 mg) alone or with LPV/r (400/100 mg). Cardiac function was monitored using continuous electrocardiograph (ECG). *Results*. Thirty-two patients were enrolled; 16 taking LPV/r -based ART and 16 ART naïve. All took single dose AL. No serious adverse events were observed. ECG parameters in milliseconds remained within normal limits. QTc measurements did not change significantly over 72 hours although were higher in LPV/r arm at 24 (424 versus 406; *P* = .02) and 72 hours (424 versus 408; *P* = .004) after AL intake. *Conclusion*. Coadministration of single dose of AL with LPV/r was safe; however, safety of six-dose AL regimen with LPV/r should be investigated.

## 1. Introduction

Malaria and HIV infection are leading causes of morbidity and mortality and remain major health problems in endemic regions. Malaria causes about 300–500 million clinical cases annually, 90% of which occur in sub-Saharan Africa [1]. The Joint United Nations Program on HIV/AIDS (UNAIDS) estimated that 29.4 Million Africans are infected with HIV (UNAIDS, December 2002). Together malaria and HIV account for over four million deaths per year. 

 Studies have demonstrated increased risk for malaria in HV infected patients especially those with lower CD4 cell counts [2–4]. More evidence suggests transient increase in HIV viral load in patients with acute malaria episodes [5]. A major challenge to the treatment of malaria in HIV-infected individuals is the potential for pharmacokinetic (PK) drug interactions with concerns regarding safety and efficacy [6]. 

Due to the widespread resistance to older antimalarial drugs, the World Health Organization now recommends artemisinin combination therapy (ACT) for malaria treatment [7]. Artemether-lumefantrine (AL) is an oral fixed-dose combination tablet of artemether (a derivative of artemisinin) and lumefantrine (a racemic mixture of a synthetic fluorine derivative). The drug combination is highly efficacious against sensitive and multidrug resistant Plasmodium *falciparum*; with the advantage of rapid clearance of parasites by artemether and the slower elimination of residual parasites by lumefantrine [7–9]. 

Recommendations for antiretroviral therapy (ART) include two nucleoside reverse transcriptase inhibitors (NRTIs) plus a nonnucleoside reverse transcriptase inhibitor (NNRTI) or a protease inhibitor (PI). Lopinavir/ritonavir (LPV/r) is an oral fixed-dose combination tablet of LPV (a PI) with low dose ritonavir, a pharmacoenhancer that significantly increases LPV plasma concentrations by cytochrome P450 3A4 (CYP3A4) inhibition. 

Concerns over safety monitoring of LPV/r have become more crucial following the recent FDA alert on cardiotoxicity of LPV/r. Safety information on LPV/r includes warnings and precautions regarding QT/QTC interval and PR interval prolongation. According to the revised safety label, LPV/r prolongs the PR interval, and cases of second- or third-degree atrioventricular block have been reported in some patients. Indeed LPV/r should be used with caution in patients who may be at increased risk of developing cardiac conduction abnormalities, such as those with underlying structural heart disease, preexisting conduction system abnormalities, ischemic heart disease, or cardiomyopathies. The effect on the PR interval of coadministration of LPV/r with other drugs that prolong the PR interval has not yet been determined and should be undertaken with caution. Clinical monitoring is recommended especially during coadministration with drugs metabolized by CYP3A [10]. 

 Data from previous studies indicate that artemether and lumefantrine are predominantly metabolized by CYP3A4 [6, 11, 12]. Knowledge of their metabolism suggests potential for PK drug-drug interactions [6]. LPV/r is a potent inhibitor of CYP3A4, therefore, inhibition of CYP3A4 may raise plasma concentrations of artemether and lumefantrine but decrease plasma concentrations of dihydroartemisinin (DHA) the metabolite of artemether. A study that investigated the pharmacokinetics of AL when administered with LPV/r in HIV-uninfected healthy volunteers demonstrated 2 to 3-fold increases in lumefantrine AUC and trends towards decreases in artemether *C*_max_ and AUC. Formal safety analysis of coadministration was not performed in this study [13]. Increased plasma concentrations of artemether and lumefantrine may enhance toxicity. Lumefantrine has some structural similarity to halofantrine which is cardiotoxic mainly in form of QTc prolongation. Therefore, vigilant evaluation of the cardiac safety of lumefantrine, especially when coadministered with a potent CYP3A4 inhibitor, is warranted [14–17]. We aimed to assess the cardiac safety of coadministration of a single dose of AL (80/480 mg) with LPV/r based ART in HIV-positive Ugandan patients.

## 2. Materials and Methods

### 2.1. Ethical Considerations

The study was approved by the Scientific Review Committee of the Infectious Diseases Institute (IDI) of Makerere University, the Uganda National HIV/AIDS Research Committee (ARC 056) and was registered with Uganda National Council of Science and Technology (HS 197) and ClinicalTrials.gov (NCT 00619944). All participants gave written informed consent to participate, and all study procedures were conducted according to Good Clinical Practice (GCP).

### 2.2. Study Site

The study was conducted between January 2008 and June 2009 at the IDI and the Uganda Heart Institute (UHI) of Mulago National Referral Hospital in Kampala, Uganda. The IDI is a regional centre of excellence for HIV/AIDS treatment, prevention, training and research. To date, over 20,000 HIV-infected patients are registered at the IDI with over 8,000 taking ART. About 10% these are on LPV/r-based second line ART.

### 2.3. Study Design and Population

This was a two-arm study to assess the safety of coadministration of AL in HIV-positive patients taking LPV/r-based ART and ART naïve patients. Patients were eligible to participate if they were older than 18 years of age, provided written informed consent, had no evidence of systemic illness and required no medications that had known potential for drug interactions with study drugs. Patients with abnormal ECG tracing, abnormal clinical test results, positive blood smear for malaria, pregnant mothers and those who reported use of herbal medication were excluded from the study.

### 2.4. Study Procedures

Patients were screened and enrolled from the cohort of patients attending the IDI. The LPV/r arm consisted of patients stable on LPV/r 400/100 mg-based ART for at least one month and the ART naïve arm consisted of patients who had not started ART and were not yet eligible for ART according to national guidelines. Patients in both arms took cotrimoxazole daily for prophylaxis against opportunistic infections. Participants had detailed study explanation at enrolment. Adherence to study drugs was assessed using self-report and pill count by the study pharmacist. We collected information on adverse drug events and serious adverse drug events, and a questionnaire on quality of life was administered on each study day. On the evening prior to the study day, participants were reminded of their study day appointment, were given detailed instructions to take their medication and food at 8:00 pm, and told to arrive at the hospital by 7:00 am in a fasting state. On the study day, patients were admitted at the UHI and a 12-lead ECG monitor was attached for continuous cardiac function monitoring. The intake of a standardized breakfast and morning doses of drugs was directly observed by study staff. All patients took a single dose of AL of 80/480 mg with 150 mL of water. Patients in the LPV/r arm took LPV/r (400/100 mg) with their AL dose. ECG monitoring was performed continuously for the first 12 hours after AL intake. Patients were then discharged and returned for the following three mornings (*T* = 24, 48, and 72 hours) for a single ECG tracing. 

### 2.5. Safety Assessment

Medical history, physical examination, vital signs, routine clinical laboratory tests, ECGs and urine screens for pregnancy were performed at screening. On the study day, medical history, physical examination, vital signs, and a blood smear for malaria parasites were performed. Adverse events were recorded continuously throughout the trial, and the onset, duration, severity, and relationship to the trial drugs if any were noted. Standard 12-lead ECGs were recorded at screening, immediately prior to dosing (*T* = 0 hour), and continuously for 12 hours after dose of AL, then daily for three days. QTc-intervals were calculated using the Bazett formula (QTc = QT/÷*√*RR) [37, 38] to correct for the influence of heart rate. A senior cardiologist evaluated the PR, QRS, and QT intervals visually on the ECG.

### 2.6. Statistical Analysis

Demographic and ECG results were entered into EpiData and exported to SPSS version 12.0 and STATA version 8.0 for statistical analysis. Continuous variables were summarized into means, and medians and compared using the Independent *T*-test. A *P*-value <  .05 was considered statistically significant.

## 3. Results

A total of 72 HIV-positive patients (41, 65% females) were screened between January and June 2009; 12 (17%) were excluded because they had other concurrent illnesses that required treatment, 28 (39%) were excluded because they had abnormal ECG tracings and 32 (44%) were enrolled, 16 in each arm. Patients in the two study arms were comparable on majority of baseline characteristics (Table 1); however, patients in the LPV/r arm had significantly higher hemoglobin levels with lower viral load. 

 There were no serious adverse events during the study period. ECG parameters (heart rate, PR-interval, QRS-complex and QTc) remained well within normal limits in both study arms (Table 2). The mean QRS-complex and QTc interval after AL administration were higher in the LPV/r arm compared to the ART naïve arm (87.4 versus 82.8, *P* = .06 and 421 versus 404, *P* = .03, resp.) but the mean PR-interval was significantly higher in the ART naïve arm (154 versus 169, *P* = .02) (Table 2). Mean (SD) change in QTc interval values from the pre-AL QTc interval values was greater for the ART naïve arm compared to the LPV/r arm (6.7 (15.4) versus −0.8 (13), *P* = .17). The QTc interval measurements for participants in both study arms remained within normal ranges over the 72 hours period (Table 3); with none above the upper limit of normal (450 ms for males and 470 ms for females).

## 4. Discussion

LPV/r is a potent inhibitor of CYP3A4, therefore, coadministration with AL which is predominantly metabolized by CYP3A4 may potentially result in enhanced pharmacological and toxicological effects. We aimed to assess the cardiac safety of coadministration of a single dose (80/480 mg) of AL in HIV-infected patients taking LPV/r based ART and HIV positive ART naïve patients. Since LPV/r is a potent CYP3A4 inhibitor, only a single dose of AL was given in order to avoid any unknown potential adverse effects of the latter. 

We found that HIV-positive patients taking LPV/r had a higher QTc interval prior to administration of AL compared to HIV-positive ART naïve patients, nevertheless, the difference was not statistically significant. It is possible that this could have been a result of the effects of LPV/r on the heart; however, we cannot establish a causal relationship since we did not have QTc measurements for these patients prior to initiation of LPV/r. This however, raises concern especially in view of the recent FDA alert over the effects of LPV/r on the heart. Indeed the label for LPV/r includes warnings and precautions regarding QT/QTc interval and PR interval prolongation [10]. 

Although the QTc interval for the LPV/r arm was significantly higher than that for the ART naïve arm at 72 hours, the difference could not be attributed to LPV/r coadministration with AL because baseline QTc interval was higher in the LPV/r arm and both study arms had an increment in QTc interval values from baseline which remained well within normal limits (Table 3). It is possible that the increment in the QTc intervals could have been more if patients had received the full six-dose regimen of AL. The LPV/r label clearly states that LPV/r should be avoided in patients using drugs that prolong the QT interval. Since we do not know what levels and effects of lumefantrine would result if the full six-dose AL regimen is coadministered with LPV/r, we suggest close clinical monitoring of HIV-positive patients taking LPV/r with AL concomitantly until more data becomes available. 

 This is one of the very few studies that have assessed the cardiac safety of coadministration of AL and LPV/r in HIV positive patients. Previous studies have evaluated safety of AL in healthy volunteers and patients with malaria. Bindschedler and others found that the QTc interval remained unchanged after a single dose of AL in healthy males [14]. The difference in results may be explained by the difference in the study populations. Bindschedler and others demonstrated significant exposure dependent increase in the QTc interval in healthy males after halofantrine. It is possible that LPV/r coadministered with a full six-dose regimen of AL may cause increased concentrations of lumefantrine causing an exposure dependent QTc interval prolongation. Previous data showed no evidence of cardiotoxicity during AL treatment in healthy volunteers [18]. However, these were conducted in patients with malaria without coadministration of LPV/r. It is possible that results may be different with concomitant treatment with the full six-dose AL regimen and LPV/r. 

## 5. Conclusion and Recommendation

Our data suggests no evidence of cardiac conduction abnormalities after concomitant treatment with LPV/r and a single dose AL. There is need to assess the safety of the full six-dose regimen of AL in HIV positive patients receiving LPV/r based ART.

## Figures and Tables

**Table 1:** It shows a comparison of the baseline characteristics of study patients.

Variable	LPV/r arm	ART naïve arm	*P* value
	median (IQR)	median (IQR)	
Age (yrs)	38 (33–41)	34 (28–39)	.2
Weight (kgs)	65 (54–73)	64 (56–71)	.9
Height (cms)	163(158–172)	163 (153–169)	.1
BMI	21 (19–24)	25 (22–31)	.06
Viral load (c/mL)	<400	26756 (5548–181186)	<.01
Hb (g/dL)	14 (14–15.4)	12.2 (12–14)	.003

**Table 2:** It shows the mean electrocardiograph (ECG) parameters in milliseconds after AL dosing.

Variable	LPV/r arm	ART naïve arm	*P* value
	mean (SD)	mean (SD)	
Heart rate	69 (8.1)	71 (5.9)	.5
PR	154 (18.4)	169 (15.9)	.02
QRS	87.4 (6.6)	82.8 (6.6)	.06
QTc	421 (20.0)	404 (20.7)	.03

**Table 3:** It shows the median QTc interval measurements in milliseconds over 72 hours period after AL dosing.

Time	QTc (ms) median (IQR)		*P* value
	LPV/r arm	ART naïve arm	
Screening	415 (403–439)	395 (388–425)	.14
12 hours	415 (404–439)	419 (403–427)	.7
24 hours	424 (401–434)	406 (393–411)	.02
48 hours	411 (396–432)	409 (401–419)	.7
72 hours	424 (416–441)	408 (392–417)	.004
